# Supplementary material for: Are carbon emissions trading and green financial instruments synergistic? -Comprehensive quantitative research based on content analysis
Source: PLoS One. 2024 Mar 7;19(3):e0298601. doi: 10.1371/journal.pone.0298601 (PMC10919601; doi:10.1371/journal.pone.0298601)
Supplement: S1 Data — (ZIP) [file pone.0298601.s001.zip › Data/地级市Z_F工作报告.zip!/║╙─╧/┬σ╤⌠╩╨/└┤╫╘╣1⁄2╓┌║┼í╛┬φ┐╦╩2╛▌═°í┐_2007.docx]

各位代表：

　　现在，我代表市人民政府向大会作工作报告，请予审议，并请各位政协委员和列席人士提出意见。

　　一、2006年工作回顾

　　2006年，是我市全面实施“十一五”规划、加快建设和谐社会的重要一年。一年来，在省委、省政府和市委的正确领导下，在市人大、市政协的监督、支持下，市人民政府团结带领全市人民顺应宏观调控，抢抓发展机遇，狠抓工作落实，圆满完成了市十二届人大三次会议确定的各项目标任务，实现了“十一五”的良好开局。

　　——综合经济实力再上新台阶。全年生产总值1331.7亿元，比上年增长15.6%，分别高出全国、全省4.9和1.5个百分点，连续16个季度保持15%以上的增速；其中一、二、三产业增加值分别增长7.5%、19.7%和10.5%；人均生产总值2.07万元。规模以上工业增加值增长26.5%，增速居全省第一。金融机构存贷款余额分别达到1058.9亿元和547.9亿元，比年初增加143.6亿元和67.6亿元。

　　——三大需求持续增长。全社会固定资产投资625.4亿元，增长37.4%；其中工业投资298.2亿元，增长42.5%。社会消费品零售总额394.8亿元，增长15.9%。进出口总额13.2亿美元，增长37.5%；其中出口7.8亿美元，增长8%。

　　——财政收支实现新突破。全部财政收入完成154.5亿元，增长27.8%；其中财政一般预算收入76.6亿元，增长25.7%。财政一般预算支出112.3亿元，增长26.4%。税收占一般预算收入的比重达到71.3%，同比提高2.8个百分点。

　　——人民生活水平显著提高。城市居民人均可支配收入和农民人均纯收入分别达到11490元和3408元，扣除物价因素，增长11.3%和15.8%。城镇居民失业登记率3.79%。居民消费价格指数上涨1.4%。城乡居民储蓄存款余额665亿元，比年初增加69.8亿元，人均储蓄首次突破万元大关。

　　(一)六大优势产业日益壮大，工业经济快速增长。大力实施项目带动。加快结构调整和产业升级，实施千万元以上工业项目484个，新开工317个，竣工311个。其中结构调整项目231个，总投资405亿元，完成投资163亿元。中信“新重机工程”、中铝洛铜10万吨电子铜板带、中硅高科2000吨多晶硅、中铝河南铝业铝板带热连轧等123个项目开工建设；一拖彪马6万台轻型汽车、华润首阳山2×60万千瓦机组、尚德30兆瓦太阳能电池、洛钼集团1万吨日选矿等124个项目竣工投产。工业经济高速增长。规模以上工业实现主营业务收入1589.2亿元，增长42.3%，高于全省平均增速4.1个百分点；实现利税总额143.7亿元、利润总额76.6亿元，分别增长47%和60.5%。黄金产量27.8万两，再创历史新高。重点企业不断壮大。全年主营业务收入超亿元企业160家，比上年增加36家，其中超200亿企业1家、超百亿企业3家、超50亿企业3家。科技创新步伐加快。新成立研发中心35家，其中国家级2家、省级3家、市级30家，争取国家、省科技项目79项、资金3184万元。列入省部共建装备制造业基地的8个“863”计划项目已完成4项。申报高新技术企业61家、产品95个。高新技术产业开发区完成技工贸总收入373亿元，实现利税32亿元。

　　(二)“三农”工作全面加强，新农村建设扎实推进。认真落实惠农政策，全年支农支出6亿多元，其中发放粮食直补和综合直补9812万元、退耕还林补助8095.6万元。落实“两免一补”政策，资助贫困学生101万人次、1.01亿元。积极推进结构调整，粮食总产量229.4万吨，再创历史新高。畜牧业快速发展，肉蛋奶总产量74万吨，农民来自畜牧业的现金收入占家庭经营现金收入的25%；烟叶、花卉苗木、林果业全面发展，六大支柱产业占第一产业的比重达70%左右。产业化水平不断提高，农业产业化龙头企业达到232家，带动农户65万户，农民来自产业化经营收入占总收入的47%。大力发展劳务经济，累计转移农村劳动力125.3万人次，劳务创收45亿元。加强农业基础设施建设，段家沟、大沟口、玉马水库除险加固工程按期完成，禹门河、金牛岭、刁崖水电站和孟西灌区建设进展顺利，前坪水库前期工作全面展开。新增有效灌溉面积3.5万亩、节水灌溉4.3万亩，治理水土流失228平方公里，改造中低产田6.6万亩。完成造林28.5万亩、通道绿化401公里。搬迁安置深山区贫困人口1.18万人。农村公益性基础设施建设步伐加快。累计投入1.5亿元，开展“村村整治、千村达标、百村示范”活动。新建农村社区服务中心600个，完成通组入户道路2612公里，解决了13.8万人的安全饮水问题；新建沼气6.4万座，我市被确定为全国沼气服务体系建设试点城市。

　　(三)旅游带动能力增强，服务业水平不断提升。旅游业发展增势强劲。成功举办第24届牡丹花会和第3届河洛文化旅游节；新安黛眉山被列入世界地质公园；游客在洛停留时间由1.5天延长到2.5天。全年接待游客2802万人次，增长30.6%；其中境外游客21.6万人次，增长56.4%。旅游门票收入3.8亿元，增长50.8%。商贸流通业快速发展。新都汇购物公园、中原康城一期等项目建成开业，新增营业面积13.75万平方米。居民消费结构升级加快，住房、汽车、通讯、电脑等高档耐用消费品大幅度增长。房地产市场日趋活跃。全年完成投资78.8亿元，增长71.1%。商品房销售323.7万平方米、61.5亿元，分别增长58.5%和74.7%。归集住房公积金5.98亿元，发放政策性个人贷款2.93亿元。

　　(四)改革开放不断深入，经济活力持续增强。国有企业改革取得新进展。洛轴集团与永煤重组成立的LYC公司生产稳定；洛玻与中建材签订了战略合作框架协议，大股东占用问题彻底解决；中铝洛铜、中钢洛耐、黄河同力、永安特钢等资产重组取得实质性进展。完成大中型企业辅业分离22家、改制13家，移交企业中小学19所，第一中医院与洛玻医院顺利合并。203户市属中小企业改革扫尾工作进展顺利。其它领域改革进一步深化。乡镇机构改革不断巩固，县乡财政体制和教育管理体制进一步理顺。农信社清收不良贷款5.2亿元。公务员登记工作基本完成，机关事业单位工资制度改革和规范津补贴工作进展顺利。深化行政管理体制和事业单位改革，清退机关借调人员和在编不在岗教师，人事编制管理进一步规范。预算管理制度改革不断深化，收支两条线、国库集中支付、政府采购等进一步加强。公路系统、公用行业改制工作基本完成。廉租房制度和住房货币化分配制度初步建立。招商引资成效显著。实际利用市外境内资金111.6亿元，增长278%；实际到位省外资金88.5亿元，增长436.4%，增幅居全省第一。新批外商投资企业48家，实际利用外资2.1亿美元，增长85.5%。非公有制经济发展势头迅猛。新上500万元以上项目618个，其中千万元以上项目399个，超亿元项目54个。非公有制企业增加值、上缴税金分别增长37.1%和31.8%，营业收入超亿元企业达到85家。

　　(五)城市建设步伐加快，城市品位明显提高。坚持规划先行，科学编制第四期城市总体规划和全市土地利用总体规划。新区建设步伐加快，全年开工各类工程400项，完成投资76亿元，累计开发25平方公里，建成道路52条83公里。隋唐城遗址植物园一期、南湖音乐喷泉等标志性工程竣工，新区体育中心、歌剧院主体完工。定鼎门污水处理厂等近30项市政公用设施建成投用。沿洛河百栋高层新开工41栋，累计开工72栋。8个村民安置小区全部竣工。城区改造稳步推进，实施改造项目150个，完成投资65亿元。定鼎北路、王城广场二期等21项工程完工，新建和改造主次干道19条、背街小巷和城中村道路334条。向阳轴承厂地块等32个开发项目完成投资50亿元。精细化管理初见成效。以创建国家卫生城市为载体，建立了市容巡查、垃圾有偿清运等一系列长效机制，对全市公厕进行改造并免费开放，实施路灯照明和景观亮化工程，城市形象更加靓丽。交通基础设施建设全面推进。全年完成投资20亿元。洛阳至南阳高速汝阳段进展顺利，庙祖线栾川方村至卢氏界、邵吉线吉利段等9条129公里干线公路建成通车，偃师-洛宁、洛阳-吉利快速通道前期工作进展顺利，改造县乡公路96.9公里、“村村通”工程2100公里，在全省提前一年实现村村通水泥路目标。县域经济发展势头强劲。县城基础设施建设加速推进，全市建制镇完成投资3.8亿元。县域经济占全市经济总量的61.5%；规模以上工业增加值增长39.8%，高于全市13.3个百分点；财政一般预算收入增长28.5%，高于全市2.8个百分点。我市有4个县(市)在全省县域经济会议上受到表彰。

　　(六)经济社会协调发展，和谐洛阳建设成效显著。就业和社会保障取得新进展。全市新增就业13.2万人，其中下岗失业人员再就业5.2万人，“4050”人员1.8万人。发放小额担保贷款9405万元。征缴各类社保基金22.2亿元，为16.6万名离退休人员增加了待遇，确保了养老金按时足额发放。城乡低保标准进一步提高，保障城市低保对象11.6万人、农村12.9万人。投入1.28亿元改造农村敬老院87所，五保户集中供养率达20.2%。在全省率先建立城乡困难群众医疗保险救助制度，参保群众27.6万人，救助资金2917万元。经济环境进一步优化。建立企业事项代理服务、企业负责人约见市领导、企业首席服务官等10项优化环境长效机制，向39家企业派出首席服务官190人次，解决重大问题52个；严肃查处破坏经济环境的违法行为。集中治理教育乱收费、公路“三乱”和医药购销、医疗服务中的不正之风，搞好食品药品、农资、成品油和文化市场整治，经济秩序进一步规范。平安建设扎实推进。坚持市领导分包信访案件、政府常务会定期听取信访汇报、党政联席会定期研究疑难案件、派驻下访工作组、启动责任调查和追究机制，一大批疑难问题得到解决，信访工作完成省定目标，信访量退出全省前10名。开展煤矿、非煤矿山、旅游、交通等安全生产专项整治，全年无重特大事故发生。深入开展平安建设十大专项活动，加强社会治安综合治理，社会大局基本稳定。社会事业全面发展。加快农村中小学布局调整，发展农村寄宿制教育，为农村中小学配备课桌凳10万余套。普通高招上省定本科线1.29万人，再创历史新高。洛阳工业高专、洛阳大学合并组建洛阳理工学院通过教育部评审。成功举办洛阳首届文化产业博览会、四国女篮对抗赛、中俄拳王争霸赛等重大活动，荣获省十运会金牌、总分第2名。加强基层文化设施建设，城乡文化事业进一步繁荣。积极参与丝绸之路申报世界文化遗产工作。乡镇卫生院国债项目、社区卫生服务中心建设进展顺利；4个试点县(市)184万人参加新型农村合作医疗，103万人享受补助5404万元；在全省率先建成覆盖城乡、功能完善、运作协调的公共卫生体系。继续稳定低生育水平，人口自然增长率控制在目标以内。实施碧水蓝天青山绿地工程，开展重点区域、重点流域环境综合整治，城市区环境空气质量优良天数303天。实施土地“三项整治”，新增、补充耕地2741.9公顷。开工建设标准化厂房52.6万平方米，建成36万平方米。民族宗教、新闻出版、统计、审计、外事、侨务、人防、地震、气象、地方志、残疾人事业等都取得了新成绩。精神文明和民主法制建设不断深入。认真实施《公民道德建设实施纲要》，深入开展社会主义荣辱观教育和形式多样的文明创建活动。双拥工作成效显著，军政军民关系更加密切，驻洛部队在支持地方经济建设、维护社会稳定中做出了重要贡献。坚持依法治市，提请市人大审议通过地方性法规4件、颁布行政规章9件。严格执行人大及其常委会通过的法规、决定和决议，自觉接受人大的工作监督、法律监督和政协的民主监督，人大代表议案、建议和政协委员提案办结率100%，满意率98%以上。强化行政执法和效能监察，严肃查处违法违纪案件，党风廉政建设取得新成绩。

　　各位代表，在宏观调控趋紧、区域竞争加剧、产业升级加快的大环境下，我们取得这样的成绩，实属不易。一年来，我们牢牢把握项目建设、招商引资、优化环境等关键环节，既适应宏观调控要求，又努力寻求发展机遇，制约发展的突出问题逐步得到解决；我们加快结构调整和产业升级，经济既保持了较高的增长速度，又保持了较好的质量和效益，发展势头更加强劲；我们加快新农村建设和农业结构调整，既注重提高农业综合生产能力，又注重解决与农民切身利益密切相关的问题，农业基础更加巩固；我们加快新区开发和城区改造，既注重建设的速度和质量，又注重文化内涵和品位，城市特色更加明显；我们在加快经济建设的同时，更加重视教育、文化、卫生、劳动和社会保障等社会事业的发展，解决了一大批关系人民群众切身利益的热点难点问题，经济社会更加和谐。河洛大地处处充满生机活力，646万洛阳人民正在中原崛起的征程上昂扬奋进！

　　各位代表，这些成绩的取得，是省委、省政府和市委审时度势、正确决策的结果，是市人大、市政协、社会各界和衷共济、全力支持的结果，是全市人民攻坚克难、锐意进取的结果。我代表市人民政府，向全市广大工人、农民、知识分子、干部，向人大代表和政协委员，向驻洛部队、武警官兵和公安干警，向各民主党派、人民团体和离退休老同志，向所有关心支持洛阳发展的各界人士，致以崇高的敬意和衷心的感谢！

　　肯定成绩的同时，还要看到经济社会发展和政府工作中仍存在不少矛盾和问题。一是结构性矛盾比较突出。农业基础依然薄弱，农民持续增收难度加大；第三产业比重比上年下降0.9个百分点，比省政府下达的责任目标低1.9个百分点；外贸出口增速较低，结构不尽合理，非公有制经济总量偏小，经济的内在活力不强。二是经济增长方式粗放。资源型、初加工工业比重高，社会化分工水平低，产业配套能力弱，产业链条短；企业自主创新能力不强，高新技术产业发展缓慢，高技术产业增加值仅占规模以上工业增加值的1.38%左右，低于全省2.15个百分点；国企改革任务依然艰巨，工业运行的质量和效益不高。三是关系民生的问题还需认真地研究、很好地解决。劳动就业、社会保障、就医就学、环境保护、食品安全、社会治安等方面还存在群众不满意的问题。四是优化环境任务依然繁重。一些部门官僚主义、形式主义严重，服务意识、执法水平和办事效率仍存在不少问题，政府自身建设还需进一步加强。对此，我们必须高度重视，采取更加有力的措施，认真加以解决。

　　二、2007年工作的总体要求

　　2007年是深入贯彻市九次党代会精神、全面走在中原崛起前列的重要一年，我们面临着新的机遇和挑战。经济全球化趋势明显，国际产业转移加快，世界经济仍处于新一轮发展的上升期。国家将加大促进中部崛起、新农村建设和和谐社会建设力度，我市及部分县被列入东北老工业基地政策和西部大开发政策享受范围，有利于争取更多的项目和资金。近年来，我市六大优势产业日益壮大，科技创新能力不断提高，综合经济实力明显增强，城市形象不断提升，为进一步加快发展奠定了坚实基础。同时也要看到，国家将继续实施稳健的财政政策和货币政策，严控两个闸门，严格市场准入，对我们加快发展、科学发展提出了更高要求。区域间竞争更加激烈，我们面临着更大的发展压力。我们既要看到自身的优势，更要正视存在的问题，抢抓一切机遇，主动应对挑战，努力加快发展。

　　今年政府工作的总体要求是：认真贯彻十六届六中全会和市九次党代会精神，以科学发展观统领全局，大力实施工业强市、旅游强市、科教强市战略，以建设经济强市、文化强市、最佳人居环境城市、最佳发展环境城市、全面发展的和谐洛阳为目标，以加快结构调整、转变增长方式为主线，以科技创新、改革开放为动力，以改善民生、促进和谐为根本，努力实现又好又快发展，在再造一个新洛阳、全面走在中原崛起前列的进程中迈出坚实步伐。

　　今年全市经济社会发展的预期目标是：生产总值增长13%；地方财政一般预算收入增长14%；全社会固定资产投资增长25%以上；社会消费品零售总额增长15%；外贸出口增长15%，实际利用外资3亿美元，利用市外境内资金150亿元；居民消费价格指数控制在3%以内；城镇居民人均可支配收入增长9%，农民人均纯收入增长6.5%；单位生产总值能耗降低4.4%；化学需氧量、二氧化硫排放量分别削减10%和20%；城镇登记失业率控制在4.5%以内；人口自然增长率控制在6.5‰以内。

　　做好今年工作，必须把握六个重点：一是更加注重结构调整和产业升级，强化农业基础地位，加快建设新型工业城市，大力发展高新技术产业，不断提高第三产业比重，努力在提高发展质量上下功夫。二是更加注重项目建设和招商引资，紧紧围绕农产品深加工、传统产业改造升级、高新技术研发和成果转化、资源合理有效开发、产品链条延伸，搞好项目谋划和前期工作，加快引进一批科技含量高、产品效益好的项目，努力在增强发展后劲上下功夫。三是更加注重城市建设和县域经济发展，不断提高中心城市的综合承载能力和辐射带动能力，提高县城和小城镇的产业支撑能力和人口集聚能力，努力在提高城镇化水平上下功夫。四是更加注重资源节约和环境保护，努力在转变增长方式上下功夫。五是更加注重服务、作风和效率，努力在优化发展环境上下功夫。六是更加注重解决好关系人民群众切身利益的问题，加强民主法制建设，捍卫公平正义，妥善处理改革发展稳定的关系，努力在促进和谐上下功夫。

　　三、加快建设经济强市和文化强市

　　突出产业主导和产业带动，促进经济与文化的相互渗透，加快经济资源大市向经济强市、文化资源大市向文化强市跨越。

　　(一)发展现代农业，促进农民增收，全面推进社会主义新农村建设。坚持把“三农”放在更加突出的位置，集中更多精力、财力和物力，加快结构调整，加强基础设施建设，促进农业全面发展、农民持续增收、农村更加和谐。进一步加大对“三农”的投入。认真落实种粮农民直补等7项补贴、农村中小学“两免一补”、计划生育奖励扶持政策。今年市级财政安排“三农”资金6755万元，比去年增加725万元。发展特色农业和品牌农业。进一步壮大六大支柱产业，全市优质小麦种植面积达到200万亩，优质玉米150万亩，粮食总产稳定在200万吨左右；加快以奶牛养殖为重点的畜牧业发展，力争畜牧业占农业总产值的比重达到35%；扩大优质烟叶、中药材、优质水果、牡丹种植面积，搞好农产品精深加工，发展订单农业，不断提高特色农产品的市场竞争力。大力推进农业产业化经营，争取市级龙头企业达到30家、省级10家，申报国家级1-2家，新发展农民专业合作组织100家以上。加快农村基础设施建设。积极推进孟西灌区、禹门河和金牛岭水电站等项目建设，做好前坪水库、伊洛河治理、中小型水库除险加固等项目前期工作，新增有效灌溉面积3万亩、节水灌溉3万亩，治理水土流失200平方公里，改造中低产田10.6万亩。搞好村镇建设规划，完善村镇服务功能，重点抓好村容村貌、路容路貌整治和社区便民服务中心、户用沼气等农村公用设施建设。

　　(二)突出项目带动，加快产业升级，努力建设新型工业城市和先进制造业基地。发展壮大以先进制造业为主的六大优势产业。全年计划实施千万元以上工业项目240个，力争实施300个，当年新开工124个，竣工120个，计划完成投资220亿元。其中安排先进制造业项目103个，总投资126亿元。重点抓好中信“新重机工程”、中铝洛铜10万吨电子铜板带、中色科技重大技术装备研制基地扩建、洛阳石化800万吨炼油扩能、中硅高科3000吨多晶硅等84个亿元以上项目建设。确保华阳孟津电厂2×60万千瓦机组、一拖工业园、伊电集团40万吨铝板带热连轧及10万吨铝型材、洛钼集团日选矿9000吨白钨回收、双瑞万基1万吨钛合金板带材、偃师ITO导电膜玻璃等124个项目按期开工建设；一拖集团3万台大中马力轮式拖拉机、洛阳尚德二期90兆瓦太阳能电池等120个项目如期竣工。加快洛阳石化45万吨PX、吉利60万吨PTA、双瑞万基2万吨海绵钛、首龙集团5万吨高精度铝箔等项目前期工作，积极谋划150万吨甲醇、100万吨二甲醚等50个项目。积极培育重点企业。引导列入省重点的14户企业和8户高成长性企业，通过资本运作实现跨越式发展。力争主营业务收入超亿元企业达到165家，超10亿元企业18家，超50亿元企业8家，超100亿元企业4家。大力发展高新技术产业，新培育高新技术企业50家、高新技术产品80个，力争高技术产业比重达到省平均水平。加快高新区发展，力争完成技工贸总收入468亿元、工业总产值360亿元。加强经济运行协调。密切关注经济走势和市场变化，重点加强煤、电、气、运、资金等生产要素的供需衔接；继续实行扭亏增盈目标责任制，搞好重点企业、亏损大户的扭亏增盈工作。加强资源节约和环境保护。建立节能降耗目标责任制，抓好冶金、煤炭、电力、建材、建筑和交通运输等领域的节能降耗工作；认真执行国家产业政策，加快淘汰普通水泥机械化立窑生产线和小火电机组；所有县(市)和吉利区污水处理厂建成投用，所有火电机组完成脱硫工程建设；继续推进龙羽宜电循环经济试点，完成豫港电力等3家电厂强制性清洁生产审核。严禁污染企业和城市污染物向农村扩散，控制农村面源污染。加快工业园区建设，引导企业向园区集聚；全年建成标准化厂房36万平方米。开展土地“三项整治”，力争盘活建设用地3万亩。

　　(三)强化旅游主导，突出商贸带动，努力提升第三产业发展水平。强化旅游业龙头作用。充分发挥历史文化优势和旅游资源优势，加大宣传推介和市场开发力度，着力打造历史文化之都、休闲度假之都和国际性旅游城市，使洛阳成为更多游客的首选地和目的地。加快西南部山区主要景点基础设施建设，努力建设伏牛山生态休闲旅游精品；重点抓好小浪底及西霞院沿黄旅游产业带的规划开发，白马寺印度风格佛殿、星级酒店、万山湖水上娱乐项目建设；大力培育生态农业、工业观光和牡丹之都等旅游品牌；精心办好第25届牡丹花会、第4届河洛文化旅游节、小浪底观瀑节和中原滑雪节。力争全年接待游客3600万人次，其中境外游客24万人次，总收入180亿元。加快商贸流通业发展。重点抓好金陵国际大酒店、建业太阳城、关林商贸城升级改造等重点项目建设，积极引入知名商业旗舰店，全力打造涧西、西工、老城和新区商业中心；加快发展金融、保险、信息、咨询、现代物流等服务业。加强社区公共服务基础设施建设，鼓励医疗卫生、商贸流通、健身娱乐等服务业向社区延伸，不断完善社区服务功能。实施“万村千乡市场工程”，扩大农村消费。加快发展生产性服务业。制定优惠政策，支持产品设计、研究开发、市场营销、售后服务等服务业加快发展，实现服务业与工业的相互促进。加快发展房地产业。规范房地产市场秩序，引导金融部门加大对住房消费的支持力度，深化住房制度改革，扩大住房公积金制度覆盖面，力争全年商品房开工700万平方米、竣工280万平方米。

　　(四)挖掘文化内涵，培育文化产业，不断增强城市文化竞争力。着力构建公共文化服务体系。做好洛阳博物馆建设前期工作；完成市区有线电视数字化整体转换；加强县级文化馆、图书馆、乡镇文化中心和文化大院建设，实施农村电影放映工程，不断丰富农村文化生活。大力发展文化产业。突出主旋律和地方特色，着力打造一批代表洛阳形象、具有较高知名度的文化精品；逐步将隋唐洛阳城等大遗址建成集保护与展示为一体的遗址公园，继续参与并推进丝绸之路、大运河洛阳段申报世界文化遗产工作；搞好民间艺术的抢救、整理和发掘，做好非物质文化遗产申报工作；大力发展牡丹书画、民间民俗文化，积极培育文化商品交易、艺术品展示、收藏、拍卖市场；鼓励和支持广告会展、文化创意和动漫制作等新兴文化产业。广泛开展体育健身、技能培训和技术服务，加快推进体育场馆、协会、彩票、俱乐部及体育中介机构产业化。深化文化体制改革，加快文化事业单位改制步伐，培育文化龙头企业，重点扶持10家有实力的民营演艺团体。

　　(五)加快科技进步，促进技术创新，不断增强可持续发展能力。今年市财政安排科技专项经费2464万元，比去年增加464万元。加强农业科技创新能力建设，完善基层农业技术推广服务体系，推进农业科技进村入户。依托骨干企业、科研院所和国内重点高校，支持列入省百户重点企业、50户高成长性企业和市属30户重点科技企业建立一批研发机构。积极支持省级多晶硅工程技术中心、先进制造技术重点实验室建设，支持河科大申报博士点，推动产学研结合，加快科技成果就地转化。加强创新人才队伍建设，培养和引进一批具有自主创新能力的高层次人才。运用高新技术产业化贴息资金、应用技术研发经费，引导企业加快关键技术和重点领域科技攻关，重点抓好多晶硅生产工艺技术与设备、海绵钛及钛合金工艺技术与设备、太阳能产业及配套技术等科技攻关，全年申报专利增长20%以上，力争进入全国科技进步先进市行列。

　　(六)统筹城乡发展，壮大县域经济，加快城乡一体化进程。积极推进偃师城乡一体化，开工建设偃师-洛宁、洛阳-吉利快速通道。加快卫星城镇建设，完善城镇功能，吸纳更多人口向非农产业和城镇转移。大力发展县域经济。围绕资源优势和产业优势，积极培育骨干财源企业，发展特色产业和主导产业，构建以工业经济为主导的产业格局，增强县域经济综合实力。力争偃师进入全国综合经济实力百强县，栾川、新安、伊川在全省前20名经济强县中位次前移，嵩县、汝阳继续保持经济发展快县地位，宜阳、洛宁、孟津进入经济发展快车道。积极发展非公有制经济。认真落实各项扶持政策，鼓励、支持非公有制经济参与国有资本调整和国有企业重组，进入公用事业、金融服务等领域。加快企业创新、创业基地和服务平台建设，完善信用担保体系，为非公有制经济提供更多的技术、融资服务。力争全年非公有制经济增加值增长16%，营业收入超亿元企业达到100家，其中超5亿元企业16家、超10亿元企业5家。

　　四、加快建设最佳人居环境城市和最佳发展环境城市

　　以建设最佳人居环境城市、最佳发展环境城市为目标，努力把洛阳建成特色突出、优势明显、独具魅力、充满活力的现代化城市。

　　(一)强化基础建设，注重生态保护，着力打造最佳人居环境城市。坚持生态环境与人文环境并重、开发建设与经营管理并重，注重科学规划，延续历史文脉，突出文化特色，体现城市个性，努力建设山水园林相间、城市功能完善、人民生活舒适、璀璨的历史文化和浓郁的现代化气息交相辉映的最佳人居环境城市。科学编制规划。加快推进第四期城市总体规划和全市土地利用总体规划修编工作。高水平做好洛河两岸风光带等一批重大项目控制性规划和详细规划，确保建设的高起点、高层次。加大新区开发力度，力争完成投资80亿元以上，房建工程累计开工面积突破1000万平方米，基本建成文化中心、体育中心和信息中心。加快南湖音乐喷泉周边地带开发，形成开元大道南北对应发展格局。加快郑西铁路客运专线洛阳南站、瀛洲桥、孙辛桥、开元大道东西延长线、新区医院等重点基础设施建设，滨河区域百栋高层累计开工110栋；积极推进新区污水处理厂、公交停车场等市政设施建设。加快洛龙科技园区建设，规划建设伊洛工业园区，增强产业支撑能力。完善商业、金融、保险、医疗、教育、文化等配套服务功能，繁荣新区服务业。大力实施城区改造，全年计划投资60亿元，实施城区改造项目150个。重点抓好金业南路、衡山路等续建工程，确保按期完工。积极做好唐宫西路、武汉路、城市东出口公交停车场和瀍河、涧河二期治理等前期工作，确保年内开工。房建开发要抓好曼哈顿广场等59个续建项目和西工步行街等91个新开工项目，确保竣工30个项目。加快交通基础设施建设。全年投资20亿元，重点抓好洛阳至南阳高速汝阳段、连霍高速洛阳东段扩建和旧邓线白河至栗树街段等8条干线公路建设。实施县乡公路改造和通村道路联网，完成县乡公路改造150公里、村村通道路1200公里。加强生态建设。完善北邙绿色屏障和城郊四大森林公园，加快环市区万亩花卉苗木产业带建设，全年新增绿地300公顷；实施飞播造林、封山育林和退耕还林，开展村庄绿化、道路绿化和农田林网建设，完成荒山造林20万亩。大力实施“五城联创”。以创建国家卫生城市为突破口，深入开展创建全国文明城市、国家环保模范城市、国家生态园林城市和中国最佳旅游城市活动，确保创卫通过国家验收。完善精细化管理评价考核体系，开展市容市貌专项整治，不断提高城市管理水平。

　　(二)扩大改革开放，优化经济环境，加快建设最佳发展环境城市。

　　深化体制改革，增强发展动力。积极探索解决发展中的深层次矛盾和问题，努力在关键环节取得新突破、重点领域取得新进展。以推进国有资本调整和国企重组引领改革。坚持政府引导和市场配置相结合，鼓励现有技术、装备、劳动力、资源等向六大优势产业集中，向有比较优势的行业和企业集中，加快培育一批具有自主知识产权、知名品牌和市场竞争力的企业集团；支持我市企业参与中央企业及其它优势企业的重组，更好地利用优势企业良好的信誉资源、信贷资源、市场资源和技术实力；继续推进洛玻与中建材的重组，围绕高档低辐射玻璃开发，实现建材玻璃向电子玻璃的跨越；支持一拖集团与中国兵装集团、洛阳凌宇公司与中集集团、白马集团与香港德生公司合作；继续深化黄河同力与省建投的合作；积极推动科研院所与企业重组，实现研发与生产相互促进，提高技术创新能力和科技成果就地转化率。以股份制改造和完善法人治理结构深化改革。利用企业战略重组、辅业改制等形式，加快推进国有企业股份制改革，逐步健全公司法人治理结构、投资风险控制机制、内部监督管理机制，做好洛轴、洛铜、洛耐、白马、黄河水泥、永安特钢等重组后的后续工作；积极争取洛轴政策性破产计划，实施春都政策性破产工作。重点扶持20家上市后备企业，加快洛钼股份、中航光电、中硅公司等7家省重点企业上市步伐。以用好老工业基地政策支持改革。积极争取洛轴等企业列入老工业基地项目支持范围，充分利用各种优惠政策，化解历史债务，分流富余人员，妥善安置职工。继续推进国有大中型企业主辅分离、辅业改制和分离企业办社会，积极做好中信重机、白马集团等7所企业中小学分离工作。以加强国资监管保障改革。完善国有企业监管制度，严格产权交易和股权转让程序，抓好清产核资、资产评估、职工安置等关键环节，防止国有资产流失，切实维护职工合法权益。深化国有资产管理体制改革，确保国有资产保值增值；加快组建市国有企业监事会，尽快向市出资企业派驻专职监事。继续做好市属203户中小企业的改革收尾工作。深入推进农村综合改革。完善乡镇机构改革，确保五年内机构、编制和财政供养人员只减不增。进一步推进农村义务教育管理体制、县乡财政管理体制和农村金融体制改革。加大农信社清欠不良贷款工作力度，确保央行票据顺利兑付。全面深化其它领域改革。稳步推进收入分配制度和事业单位人事制度改革，确保机关事业单位工资和津补贴发放；深化金融体制和投融资体制改革，加快城市管理、干线公路投融资、县乡公路养护体制改革。

　　积极扩大开放，增强发展活力。进一步提高招商引资水平。完善领导带队招商、招商奖励等制度，创新招商方式，拓宽招商渠道。积极策划500个招商项目，重点面向国内外知名企业搞好推介。精心筹备第25届牡丹花会、第2届中博会洛阳分会场和豫商大会招商工作。重点做好签约项目的跟踪服务，切实提高合同履约率、资金到位率、项目开工率。大力发展资本市场，加快融资步伐，提高金融保险、公用事业、基础设施建设等领域的开放水平，吸引更多的金融保险机构来洛。优化进出口商品结构，巩固扩大传统大宗产品出口，积极培育一批科技含量高、附加值高的新型出口企业，不断提高机电产品和高新技术产品的比重。鼓励有条件的企业开展跨国经营，参与国际合作与竞争。积极开展对外工程承包与劳务合作，不断增强经济发展的外向度。

　　优化经济环境，增强对发展要素的吸引力。认真落实市领导联系企业、企业首席服务官、企业直通车、行政执法绩效考核等10项制度，不断完善优化经济发展环境的长效机制。规范行政审批行为，减少审批事项，增加核准和备案事项，建立网上审批制度，完善一站式办公、一条龙服务。坚持双休日正常办理请办事项制度、企业反映突出问题周报制度；加强执法监督，严禁对企业重复执法、无备案执法、无证执法、粗暴执法、“安静生产日”乱检查、无审核收费罚款；坚持对企业首次不罚、重大行政处罚听证、对企业停产等处罚事项市政府审批制度。认真受理企业和群众投诉，对破坏经济环境的行为要严肃查处，公开曝光，决不手软。努力营造负担最轻、秩序最好、办事最快、信誉最好、纳税人最满意的发展环境。

　　五、加快建设全面发展的和谐洛阳

　　更加关注民生民情，切实解决好教育、医疗、就业和社会保障等群众最关心、最直接、最现实的利益问题，让人民群众享受到更多的发展成果。

　　(一)全力办好市委、市政府承诺的20件实事。要为农民办好十件实事。一是新建农村户用沼气20万座，新发展沼气技术服务网点80家；二是解决20万人的饮水安全问题，保证群众喝上干净水、放心水；三是完成200个示范村、300个重点整治村通组入户道路建设，硬化道路3000公里；四是农村低保对象做到应保尽保，补差标准由每人每月20元提高到30元。新建和改扩建乡镇敬老院50所，五保对象集中供养率达到30%；五是全面推行新型农村合作医疗制度，继续实施农村困难群众补充医疗保险救助制度，解决425万农民群众看病难问题；六是全部免除农村义务教育阶段学生学杂费，继续对困难家庭学生免费提供课本并补助寄宿生生活费，更新农村学校课桌凳，改善农村贫困学校寄宿生饮食条件；七是对居住在深石山区的3000户1.2万人实施扶贫搬迁，改善贫困山区群众生存环境；八是对20万名农村劳动力进行职业、劳务技能和实用技术培训，完成农村劳动力转移125万人以上；九是完成822个村3.4万户广播电视“村村通”任务，建成市级无线数字电视中心机站；十是在全市50个乡镇建设乡级便民服务中心，在距乡镇政府所在地较远的行政村设立便民服务点，抓好600个行政村的社区服务中心建设。要为城镇居民办好十件实事。一是完成8条城市道路和七一南路雨水泵站建设任务，支持城市区281条背街小巷改造。市区和新区各增加1条公交线路，优化调整2条线路，新购置100辆环保公交车，双休日、节假日增加开往旅游景点的车辆；二是落实廉租住房管理办法，缓解市区1200户“双困”家庭的住房困难；全年竣工经济适用房20万平方米，解决2200余户群众住房困难；三是新增就业10万人，实现下岗失业人员再就业3.47万人，安排“零就业”家庭每户1人就业；四是继续实施城市困难群众补充医疗保险救助制度，缓解15万名城市困难群众看病难问题；建设44个社区卫生服务中心、70个社区卫生服务站，方便居民就近就医；五是新增城镇基本养老保险参保人数2.5万人、城市低保对象2万人以上，城镇基本医疗保险参保人数达到70万人以上；六是消除城市区中小学D级危房，全部免除城市低保家庭子女义务教育阶段学杂费，保证进城务工人员子女入学；七是加大大气、水污染治理力度，全年削减二氧化硫排放量10万吨，确保城市空气良好以上天数稳定在300天以上；八是创建国家级商业示范社区1个，省级商业示范社区7个，在部分社区构建10分钟商业网点服务体系，满足居民基本生活需要；九是实施洛浦公园东延伸工程和隋唐城遗址植物园二期工程，在洛北城区新增4万平方米休闲广场；十是组织对5000人次社区居民进行体质监测；为每个新建社区配备6件以上健身路径；新区体育设施适时向市民免费开放；组织全市性市民健身活动20次以上、比赛3次以上。

　　(二)加快发展社会事业。加大基础教育投入，全年市财政安排教育专项经费6542万元，比去年增加1027万元。稳步推进中小学布局调整，改善农村中小学基础条件，加快边远山区寄宿制学校建设，不断增强农村义务教育保障能力；启动第二批农村现代远程教育工程，力争年内覆盖所有中小学。加强市区薄弱学校建设，下大力解决部分学校班额过大问题。加快职业教育资源整合，努力为经济发展培养更多实用技术人才。支持高等院校重点学科建设，办好洛阳理工学院。建立公共卫生体系长效管理机制，做好重大疾病防控；加快村级标准化卫生所建设，完善城市社区卫生服务网络。加强医院管理，提高医疗服务水平。实施食品药品放心工程，保障人民群众饮食用药安全。继续稳定低生育水平，提高出生人口素质。加快市残疾人综合服务中心建设，努力做好残疾人就业和社会保障等工作。加强民族团结，依法管理宗教事务。继续做好统计、审计、外事、侨务、人防、新闻出版、气象、地震、地方志等工作。

　　(三)全面加强社会保障。全年市财政安排社会保障和就业支出11265万元，比去年增加4980万元。深化企业养老保险制度改革，降低企业缴费费率，逐步做实个人账户；做好社会保险扩面征缴清欠工作，将非公有制企业、灵活就业人员、农民工和失地农民纳入覆盖范围；妥善解决市属破产、撤销和困难企业退休人员医疗保险问题，积极争取全国城镇居民基本医疗保险制度改革试点；实现失业保险市级统筹。合理调整和严格执行最低工资标准，严肃查处损害劳动者合法权益的案件，积极构建和谐劳动关系。认真解决老国有企业职工住房困难问题。发挥商业保险在健全社会保障体系中的作用，积极争取国家农业政策性保险试点。鼓励社会力量兴办养老机构，大力发展社会福利事业和慈善事业。

　　(四)认真做好财税工作。依法加强税收征管，全面清理和规范非税收入，不断提高税收占财政收入的比重，努力做到应收尽收。深化财税体制改革，加快公共财政体系建设，完善国库集中支付制度，规范政府采购行为。调整财政支出结构，确保农业、教育、科技、社会保障等支出高于去年。

　　(五)努力建设平安洛阳。深入开展平安创建活动，完善社会治安防控体系，严厉打击各种刑事犯罪活动。发挥洛阳驻军和民兵预备役在平安建设中的作用，努力形成民警民兵共同维护社会治安的格局。强化基层基础建设，开展矛盾纠纷排查化解年活动，解决好涉及群众切身利益的问题，争创全省平安建设先进市。严格安全生产责任制，健全重大危险源和重大事故隐患排查、整改、消除机制，坚决遏制重特大事故发生。建立健全应急救援体系，有效应对自然灾害、事故灾难、公共卫生、食品药品和社会安全事件，维护社会大局稳定。

　　(六)加强精神文明建设。加强爱国主义、集体主义、社会主义教育，大力弘扬新时期洛阳人民的创业精神。扎实开展道德规范进万家、诚实守信万人行活动和文明城市、文明景区创建活动，着力打造诚信洛阳、礼仪洛阳、魅力洛阳。坚持党政机关包村扶贫和支持新农村建设，开展市直部门联系社区、公务员走访慰问困难户活动。加强国防教育，积极支持驻洛部队和民兵预备役建设，继续做好双拥工作，巩固和发展军政军民团结的大好局面。

　　六、加强政府自身建设

　　新的形势、新的任务、新的征程，要求我们必须加强能力建设，改进工作作风，以饱满的精神、昂扬的斗志，团结带领全市人民，求真务实，不畏艰难，全力以赴，加快发展。

　　(一)加快建设创新型政府。牢固树立科学发展观和正确的政绩观，加强学习，勤于思考，不断丰富理论素养，提高执政水平。紧紧围绕经济社会发展的新形势、新实践，研究政策、吃透政策、运用政策，努力谋求更好更快发展。推进体制创新、机制创新和管理创新，改进管理与服务方式，确保权力运行公开透明。

　　(二)加快建设服务型政府。强化社会管理和公共服务，逐步建立科学有效的利益协调机制、诉求表达机制、矛盾调处机制和权益保障机制。加强电子政务建设，推进政务公开和公共服务信息化。完善绩效考核制度，全面推行服务承诺制、首问负责制、限时办结制，高效服务，优质服务。同时，建立责任调查追究机制，不断提高行政效能。

　　(三)加快建设法治政府。坚持依法治市，深入开展普法教育，提高全民法律素质。认真落实《监督法》，及时向人大、政协报告工作、通报情况，自觉接受人大及其常委会的法律监督、工作监督和政协的民主监督，严格执行人大及其常委会的决议、决定，支持人大代表依法行使职权。健全重大事项集体决策、专家咨询、社会公示与听证、决策评估制度，推进决策民主化、科学化。认真办理人大代表议案、建议和政协委员提案，广泛听取各民主党派、工商联和无党派人士的意见和建议，充分发挥人民团体的桥梁纽带作用，发挥群众监督和舆论监督作用，做到察民情、听民声、集民智、顺民意。加强政府立法，强化执法监督，推行执法责任制，依法受理行政复议案件。搞好法律援助，依法保障公民特别是妇女、未成年人和残疾人的合法权益。

　　(四)加快建设廉洁政府。建立健全教育、制度、监督并重的惩治和预防腐败体系。严格建设工程招投标、土地拍卖挂牌出让、政府采购、企业国有资产交易等制度，加强对权力运行的制约和监督。开展商业贿赂专项治理，坚决纠正损害群众利益的各种不正之风，严肃查处违法违纪案件。深入开展“讲正气、树新风”活动，教育广大公务员严以律己，秉公用权，廉洁从政。

　　(五)加快建设诚信政府。加强诚信教育，提高公务员的责任意识，建设一支高素质的公务员队伍。建立失信惩戒机制、责任追究机制和赔偿机制，确保政府及其工作人员忠实履行职责。落实对企业和外来投资者的优惠政策，兑现对人民群众的承诺，建设人民满意政府。

　　各位代表！做好今年政府工作，顺利实现预期目标，对再造一个新洛阳、全面走在中原崛起前列具有重要意义。蓝图绘就，任重道远，责任如山。让我们高举邓小平理论和“三个代表”重要思想伟大旗帜，抢抓机遇、加快发展、科学发展、和谐发展，为创造洛阳更加美好的未来而努力奋斗！
